# Effect of BIX-01294 on H3K9me2 levels and the imprinted gene *Snrpn* in mouse embryonic fibroblast cells

**DOI:** 10.1042/BSR20150064

**Published:** 2015-10-19

**Authors:** Peng Chen, Jian-Feng Yao, Rong-Fu Huang, Fang-Fang Zheng, Xiao-Hong Jiang, Xuan Chen, Juan Chen, Ming Li, Hong-Feng Huang, Yi-Ping Jiang, Yan-Fang Huang, Xiao-Yu Yang

**Affiliations:** * The First Affiliated Hospital, Fujian Medical University, Fuzhou, PR China; † College of Preclinical Medicine, Fujian Medical University, Fuzhou, PR China; ‡ The Second Affiliated Hospital, Fujian Medical University, Quanzhou, PR China; § Quanzhou Medical College, Quanzhou, PR China; ║ Institutes of Reproductive Sciences, Fujian Medical University, Fuzhou, PR China; ¶ The Affiliated Fuzhou First Hospital, Fujian Medical University, Fuzhou, PR China; ** Fuzhou Maternity & Child Healthcare Hospital, Fuzhou, PR China

**Keywords:** diazepin-quinazolin-amine derivative (BIX-01294), cellular toxicity, DNA methylation, histone dimethylation, mouse embryonic fibroblasts, *Snrpn*

## Abstract

Histone H3 lysine 9 dimethylation (H3K9me2) hypermethylation is thought to be a major influential factor in cellular reprogramming, such as somatic cell nuclear transfer (SCNT) and induction of pluripotent stem cells (iPSCs). The diazepin-quinazolin-amine derivative (BIX-01294) specifically inhibits the activity of histone methyltransferase EHMT2 (euchromatic histone-lysine *N*-methyltransferase 2) and reduces H3K9me2 levels in cells. The imprinted gene small nuclear ribonucleoprotein N (*Snrpn*) is of particular interest because of its important biological functions. The objective of the present study was to investigate the effect of BIX-01294 on H3K9me2 levels and changes in *Snrpn* DNA methylation and histone H3K9me2 in mouse embryonic fibroblasts (MEFs). Results showed that 1.3 μM BIX-01294 markedly reduced global levels of H3K9me2 with almost no cellular toxicity. There was a significant decrease in H3K9me2 in promoter regions of the *Snrpn* gene after BIX-01294 treatment. A significant increase in methylation of the *Snrpn* differentially methylated region 1 (DMR1) and slightly decreased transcript levels of *Snrpn* were found in BIX-01294-treated MEFs. These results suggest that BIX-01294 may reduce global levels of H3K9me2 and affect epigenetic modifications of *Snrpn* in MEFs.

## Abstract (precis)

BIX-01294 (a diazepin-quinazolin-amine derivative) has important biological effects and its epigenetic regulation at imprinting control regions is highly complex. BIX-01294 may reduce global H3K9me2 levels and affect epigenetic modifications of small nuclear ribonucleoprotein N (Snrpn) in MEFs.

## INTRODUCTION

Studies have shown that somatic cell nuclear transfer (SCNT) embryos consistently display aberrant histone H3 lysine 9 dimethylation (H3K9me2) hypermethylation [1]. The sequence of events in the induction of pluripotent stem cells (iPSCs) by defined factors is poorly understood. Chen et al. [2] showed that H3K9 methylation is a barrier to somatic cell reprogramming into iPSCs, indicating that H3K9me2 hypermethylation might be a major influential factor in embryonic reprogramming. This hypothesis is supported by Sridharan et al.’s [3] demonstration that inhibition of H3K9 methylation enhances reprogramming.

Euchromatic histone-lysine *N*-methyltransferase 2 (EHMT2, also known as G9a) is believed to be dominant in catalysing H3K9me2 and regulates gene repression [4]. It has been suggested that EHMT2-mediated gene silencing is a major factor involved in the mechanism of embryonic reprogramming [3]. The levels of H3K9me2 increase as a result of the increased histone methyltransferase EHMT2 activity. Furthermore, H3K9me2 levels can be reduced by specific inhibition of the EHMT2 activity in cultured cells.

The diazepin-quinazolin-amine derivative (BIX-01294) is a small molecule compound that specifically inhibits EHMT2 enzymatic activity and reduces H3K9me2 levels in the chromatin regions of several EHMT2 target genes [5,6]. Its beneficial role in enhancing somatic cell reprogramming has been confirmed previously [7]. Depletion of the H3K9 methyltransferase EHMT2 increases iPSC formation from both fibroblasts and late reprogramming intermediate (pre-iPSCs) [3]. Therefore, BIX-01294-treated donor cells with down-regulated H3K9me2 levels may ultimately improve the efficiency of iPSC generation. However, previous studies have shown that BIX-01294 has cellular toxicity [8,9], indicating that high concentrations of BIX-01294 may affect the growth or even kill cells.

Fibroblast cells are a convenient resource for biochemical studies and are the most common donor cells in SCNT technology, as well as being important donor cells in the generation of iPSCs. In the present study, we used mouse embryonic fibroblasts (MEFs) to investigate the effect of BIX-01294 on H3K9me2 levels and to determine the optimal concentration of BIX-01294 required to reduce H3K9me2 levels in MEFs with no cellular toxicity. This information will provide an experimental basis for the use of BIX-01294 in research involving MEFs.

Investigations of the changes in the imprinted gene small nuclear ribonucleoprotein N (*Snrpn*) gene are crucial for probing the molecular mechanisms of the neurodevelopmental disorder, known as Prader–Willi (PWS) and Angelman syndromes (AS). The PWS imprinting centres (PWS-IC) in human and mouse contains the promoter region of the imprinted *Snrpn* gene [10]. The maintenance of cytosine-phosphate-guanosine (CpG) methylation of the PWS-IC in mouse ES cells requires the function of histone methyltransferase EHMT2 [11]. The mouse *Snrpn* gene, a maternally imprinted gene, encodes the survival of motorneurons protein (Smn protein), which is involved in RNA splicing. It maps to mouse chromosome 7C, which is homologous to human chromosome 15q11–q13 [12]. The imprinting features are conserved between mice and humans. *Snrpn* is of particular interest because of its important biological functions and the characteristic phenotypes associated with the absence of its gene product. Human *SNRPN* has been reported to be methylated in central neurocytomas and in paediatric germ cell tumours (GCTs), being inactivated with hypothesized tumour suppressor function of the specific gene [13]. The imprinted transcript of *Snrpn* is present in MEFs [14]. In the present study, we analysed epigenetic modifications and changes in the transcript levels of imprinted *Snrpn* gene in BIX-01294-treated MEFs.

## MATERIALS AND METHODS

### Establishment of embryonic fibroblasts and culture maintenance

All chemicals were purchased from Sigma—Aldrich, unless otherwise noted. Experiments were approved by the Ethics Committee on Animal Experiments of Fujian Medical University (China). Primary cultures of embryonic fibroblasts were established from fetal mice at 14.5 days post-coitum (dpc) and three different mice were used for obtaining MEFs. The body of the fetal mouse was isolated from the head, tail, limbs and internal organs and used for primary cultures of MEFs, adopting the tissue-piece cultivation method [15]. Cultures were passaged by releasing cells with trypsin and re-seeded at an initial concentration of 100000 cells/25 cm^2^ flask. At 80% confluence, the culture medium of Dulbecco's modified Eagle medium (DMEM)/F12 containing 10% fetal calf serum was replaced with a fresh medium supplemented with BIX-01294. Studies [6] showed that BIX-01294 had an IC_50_ of 1.7 μM *in vitro*, so firstly we estimated cytotoxicity of BIX-01294 at various concentrations (0, 1, 1.3, 1.7, 2, 2.2, 3 or 4 μM). After being cultured in the BIX-01294 containing medium for a further 24 h, cells were collected for further analysis.

### MTT assay

Cytotoxicity of BIX-01294 at various concentrations was estimated using MTT assays as previously described [16]. Briefly, MEFs in the logarithmic growth phase were seeded at a concentration of 1.5×10^4^ cells/ml (100 μl) into a 96-well plate for 24 h. BIX-01294 was added to four wells in each group. The cells were cultured at 37°C under 5% CO_2_ for 24 h. The culture medium was removed and 20 μl of MTT was added to each well for a 4-h culture. The medium was removed and DMSO was added to dissolve the crystals. By using the Microplate Reader (Bio-Rad 680), the absorbance of the solution at 492 nm (*A*_492_) was measured and the relative growth rate (RGR) of cells was calculated as follows: RGR (100%)=*A*_E_/*A*_C_ × 100%, where *A*_E_ is the absorbance in the experimental group and *A*_C_ is the absorbance in the control group [16].

### Immunocytochemical analysis of global histone H3K9 dimethylation

Primary antibodies against H3K9me2 (Abcam) and secondary antibodies [FITC-conjugated goat anti-mouse IgG (Millipore)] were used for immunostaining. MEFs attached to coverslips were washed with PBS–Tween (0.05% Tween-20 in PBS), fixed in 4% paraformaldehyde in PBS for 15 min and permeabilized with 0.2% Triton X-100 in PBS–Tween at room temperature (RT) for 15 min. The cells were further treated with 2 M HCl at RT for 30 min and subsequently washed in PBS–Tween, prior to incubation in blocking solution (2% BSA in PBS–Tween) for 60 min at RT. Cells were then incubated overnight at 4°C with the primary antibody diluted 1:200 in blocking buffer. The cells were washed three times for 5 min in PBS–Tween and incubated for 1 h at RT with the secondary antibody, diluted 1:400 in blocking buffer. DNA was stained with 5 mg/ml Hoechst 33342 for 2 min. The cells were washed three times for 5 min in PBS–Tween and mounted on to glass slides. Cell samples were examined using a confocal microscope (Leica TCS SP5). The experiment was repeated three times and normal serum without primary antibody was used as a negative control. Leica Microsystems software (LAS AF Lite Version: 1.8.1 build 1390) was used for quantitative analysis and the methylation status was calculated as the intensity of FITC staining divided by the intensity of nuclear staining with Hoechst 33342 [15].

### Western blots [8]

Primary antibodies against H3K9me2 (Millipore) and secondary antibodies [HRP-conjugated goat anti-mouse IgG (Pierce)] were used. After being cultured in 35 mm dishes for 2 days and reaching 90% confluence, cells were lysed with 60 μl of lysis buffer (Beyotime) and mixed with 15 μl of loading buffer. Protein samples were boiled for 10 min (for denaturation) and subjected to SDS/PAGE (12% gel), before transfer on to nitrocellulose filter (NC) membranes (Whatman). The NC membranes were blocked with 5% non-fat dried milk at 4°C overnight, incubated with primary antibody diluted 1:2000 at RT for 1 h and washed three times with 0.58 g/l NaCl, 0.05% Tween 20 and 20 mM Tris/HCl. Membranes were then incubated with secondary antibody diluted 1:2000 at RT for 2 h. After three washes, NC membranes were treated with super ECL Plus (Applygen) and then the western blot signals were detected by exposure to films. Signal intensities were quantified using Image J 1.37v software (National Institutes of Health).

### Real-time reverse transcription PCR (RT-PCR)

MEFs cultured in DMEM/F12 medium and in BIX-01294 containing medium (designated BB and AB cells respectively) were used for RNA extraction using the Trizol reagent (Bio Basic Inc., BS410) according to the manufacturer's protocol. cDNAs were generated from total RNA with ReverTra Ace-a-TM (TOYOBO) primed oligo (dt) 20 primers. Quantitative analysis of each cell sample was carried out using SYBR RT-PCR kits (Takara) and the ABI Prism 7500 system (Applied Biosystems). Two-step quantitative RT-PCR (qRT-PCR) of double-curve standards was performed on triplicate samples to control for PCR variations according to the manufacturer's protocol. Each experiment was performed three times on independent RNA extracts from three different mice. The quantity of each cDNA sample measured was normalized to the reference gene *β-actin*. Primers for amplification of *Snrpn* and *β-actin* transcripts were designed by Sangon Biotech and are listed in Table 1. Single product amplification was verified by melting curve evaluation and electrophoresis of qRT-PCR products on 1% agarose gels. The PCR efficiency for each primer pair was determined using 5-fold serial dilutions of cDNA transcripts. The linear correlation coefficient (*R*^2^), which is an indicator of the goodness-of-fit of the standard curve, was plotted for the standard data points of all genes; these values ranged from 0.984 to 0.998. Cycling conditions were 95°C for 10 s, followed by 40 cycles at 95°C for 5 s, then 60.5°C for 34 s.

**Table 1:** Primer pairs used in real-time PCR and DNA methylation

Function(s) and name	Primer sequence5′–3′	Product size
Transcription analysis		bp
*Snrpn-Q-F*	AGGCCCATCCCAGCAGGTCAT	118
*Snrpn-Q-R*	GCGGGTACTGGGTTGGGGCTC	118
*β-actin-Q-F*	TCCATCATGAAGTGTGACGT	135
*β-actin-Q-R*	GAGCAATGATCTTGATCTTCAT	135
Methylation analysis		
Mellst-F	TATGTAATATGATATAGTTTAGAAATTAG	533
Mellst-R	AATAAACCCAAATCTAAAATATTTTAATC	533
Mel2nd-F	AATTTGTGTGATGTTTGTAATTATTTGG	420
Mel2nd-R	ATAAAATACACTTTCACTACTAAAATCC	420
ChiP-QPCR analysis		
Q-ChiP-F	ACACGCTCAAATTTC	130
Q-ChiP-R	TAGTCTTGCCGCAATGGCTC	130

### DNA methylation analysis of *Snrpn*

The AF081460 (GenBank) sequence was used for DNA methylation analysis. DNA isolated from MEFs was treated with sodium bisulfite using a CpG genome DNA modification kit (Chemicon International Inc.), according to the manufacturer's protocol. Bisulfite-treated DNA samples were subjected to nested PCR amplification of differentially methylated region 1 (DMR1). The initial PCR was carried out with the first primer set listed in Table 1. The amplification products were diluted (100×) and used as templates for nested PCR using the second primer set (Table 1). The nested PCR products were cloned into the TA cloning vector (pMD18-T) and at least 10 clones for each sample were sequenced.

### ChIP

A ChIP assay was performed with the EZ-ChIP assay kit (Millipore) according to the manufacturer's protocol. The detailed description is available in our previous report [15]. Antibodies against H3K9me2 were obtained from Abcam Biotechnology. Samples immunoprecipitated with anti-histone H3 antibody and immunoglobulin G were used as positive and negative controls respectively. For each of the precipitated samples, the ratio of immunoprecipitation was determined by comparison with the input chromatin, represented by the total DNA in each sample before the addition of anti-H3K9me2 antibodies.

### Quantitative analysis of immunoprecipitated DNA by real-time PCR

Immunoprecipitated DNA and input DNA were analysed quantitatively by real-time PCR using a previously described protocol [17]. Primers are listed in Table 1. Each PCR was run in triplicate (at least) to control for PCR variation and each experiment was repeated three times. To compare histone modifications, the amount of immunoprecipitated DNA was normalized by dividing the average value of each histone modification by that of the internal control, *β-actin*. Each normalized value of the immunoprecipitated DNA was further divided by the normalized value of the corresponding input DNA. BB and AB cells were compared by calculating the percentage immunoprecipitation according to the average value of immunoprecipitated DNA divided by the average value of the corresponding input DNA [15].

### Statistical analysis

Variations in the levels of *Snrpn* mRNA, the relative levels of methylated DNA and dimethylated histone H3K9 for MEFs cultured in DMEM/F12 medium and in BIX-01294-containing medium were calculated using the SPSS Ver.13.0 software. Differences among treatment groups were analysed by Student's *t*tests, with *P*<0.05 considered to indicate significance.

### RESULTS

#### Estimation of BIX-01294 cytotoxicity on cultured MEFs

The cultured MEFs became sparse and exhibited anomalous morphology after incubation with BIX-01294 at 2 μM or higher, indicating that BIX-01294 is cytotoxic to MEFs at these concentrations. Furthermore, based on the RGR (Table 2) [18], MTT assays also indicated that 2 μM BIX-01294 (RGR=78.14%) or higher produced mild cytotoxicity in cultured MEFs (Figure 1). MEFs of treatment with BIX-01294 at 0, 1.0, 1.3, 1.7 and 2.2 μM for 24 h were analysed by confocal microscopic evaluation of immunocytochemical staining. The cell morphology was apparently normal when treated with BIX-01294 at 1.3 μM or lower, although the nucleus was reduced in size at 1.7 and 2.2 μM (Figure 2A). In summary, BIX-01294 showed almost no MEF cytotoxicity at concentrations of 1.3 μM or lower.

**Table 2:** Cytotoxicity evaluation reference standards

RGR	Grade	Cytotoxicity
>100%	Grade 0	No
80%–99%	Grade 1	No
50%–79%	Grade 2	Mild
30%–49%	Grade 3	Moderate
0%–29%	Grade 4	Obvious

**Figure 1:**
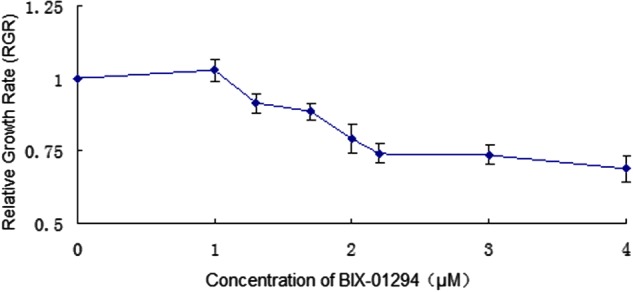
Effect of BIX-01294 on MEF RGR measured using MTT assays

**Figure 2:**
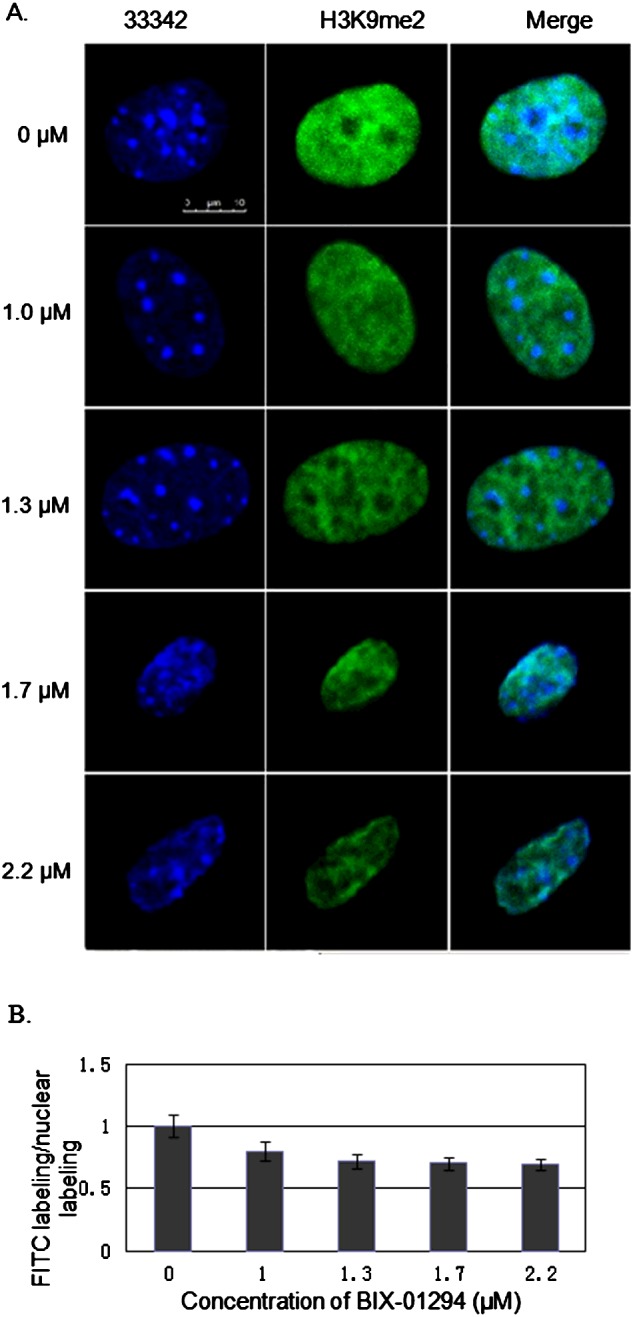
Global histone H3K9me2 in cultured MEFs analysed by immunocytochemistry The level of global H3K9me2 was detected by binding of FITC-labelled antibodies specific to H3K9me2. (**A**) Visualization of H3K9me2-immunostained fibroblasts cultured in DMEM/F12 medium and in medium containing BIX-01294 at various concentrations. Green: FITC-labelled H3K9me2; blue: Hoechst-stained nuclei. Scale bar=10 μm. (**B**) Dimethylation status was calculated as the intensity of FITC labelling divided by the intensity of nuclear labelling with Hoechst 33342.

### Effect of BIX-01294 on H3K9me2 levels

Immunostaining showed a small but statistically significant reduction in H3K9me2 staining intensity in the groups treated with BIX-01294 between 0 μM and the other densities (1.0, 1.3, 1.7, 2.2 μM; *P*<0.05), whereas there was no statistically significant difference among the groups treated with the higher concentrations (1.0, 1.3, 1.7, 2.2 μM; *P* > 0.05; Figure 2). Finally we concluded that 1.3 μM BIX-01294 was the maximum concentration that induced changes in global histone H3K9me2with negligible cytotoxicity. Moreover, western blot analysis was used to confirm that 1.3 μM BIX-01294 could significantly reduce H3K9me2 indeed. (BB 0.761±0.089, AB 0.310±0.075, *P*=0.016; Figure 3). So we selected 1.3 μM BIX-01294 for further analysis.

**Figure 3:**
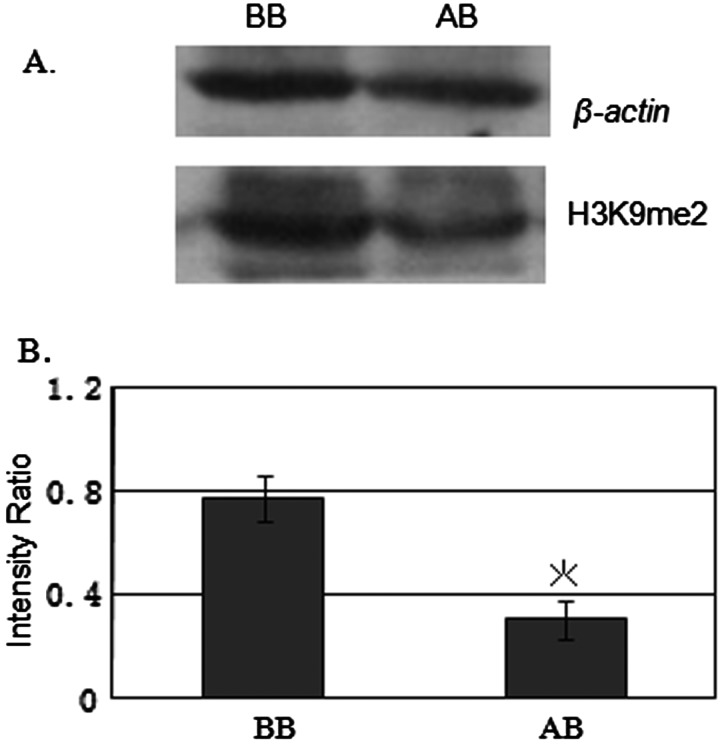
Global histone H3K9me2 in cultured MEFs analysed by western blotting (**A**) Western blot analysis of H3K9me2 in MEFs incubated with 1.3 μM BIX-01294. *β-Actin* served as a loading control. (**B**) Quantification of the intensities of western blot signals. Intensity ratios represent the signal intensity of H3K9me2 relative to that of *β-actin.* The analysis was repeated three times and the quantitative value is expressed as mean ± S.E.M. The asterisk (*) indicates a significant difference between BB and AB (MEFs cultured in DMEM/F12 medium and in BIX-01294-containing medium respectively; *P*<0.05).

### Reduction in *Snrpn* transcript levels in BIX-01294-treated MEFs

Real-time fluorescent quantitative PCR analysis of *Snrpn* transcript levels in MEFs treated with and without BIX-01294 (AB and BB cells respectively) revealed a small but statistically significant reduction in the transcript levels in AB cells compared with those in BB cells (Figure 4; BB 1.7890±0.0584, AB 1.5125±0.0375, *P*=0.020).

**Figure 4:**
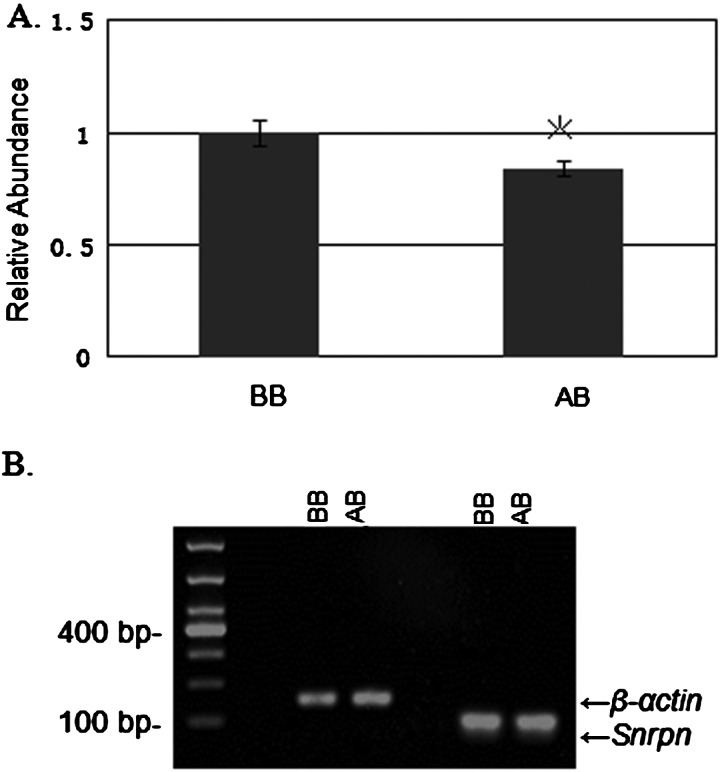
*Snrpn* transcripts in cultured MEFs analysed by real-time PCR using SYBR green (**A**) The relative abundance of *Snrpn* mRNA transcripts in AB was calibrated against those in BB. The quantity of each cDNA sample measured was normalized to the reference gene *β-actin*. MEFs are from three different mice and data are expressed as mean ± S.E.M. (indicated by bars). The asterisk (*) indicates a significant difference between BB and AB (MEFs cultured in DMEM/F12 medium and in BIX-01294-containing medium respectively; *P*<0.05). (**B**) Agarose gel electrophoresis of real-time PCR products.

### Histone H3K9 dimethylation of *Snrpn* decreased in cultured MEFs

ChIP assays were performed on cultured embryonic fibroblasts treated with and without BIX-01294 (AB and BB cells respectively) using specific antibodies against H3K9me2. Each value was normalized against *β-actin* as an internal control. Dimethylated H3K9 histones were clearly immunoprecipitated in both BB and AB cells. Furthermore, the results of ChIP were presented as percentage immunoprecipitation using ‘%Input’ values to compare BB and AB cells. Unmodified histone H3, which is present in all nucleosomes, was used as positive control. Non-specific IgG, which served as a negative control, showed very low enrichment values (between 0.0005% and 0.0042%) of non-specific binding to the promoter regions of the *Snrpn* gene. Compared with the BB cells, there was a significant decrease in H3K9me2 in the AB cells (BB 1.000±0.093, AB 0.600±0.079, *P*=0.000).

### DNA methylation of DMR1 increased in cultured MEFs

*Snrpn* contains two DMRs. DMR1 is thought to be a germline DMR and contains the *Snrpn* promoter [19]. We analysed the methylation status in MEFs treated with and without BIX-01294 (AB and BB cells respectively) within the 6-kb 5′-DMR1 sequence, including a portion of the promoter and the transcription start site of *Snrpn*, using the bisulfite method. The *Snrpn* promoter is differentially methylated in MEFs at this position. For each sample, 10 clones were sequenced and 16 CpG sites were detected in DMR1. Analysis of the methylation status of DMR1 in BB and AB cells showed that the methylation increased significantly after treatment with BIX-01294 (65.62% in AB compared with 37.50% in BB, χ^2^=25.337, *P*<0.000; Figure 5).

**Figure 5:**
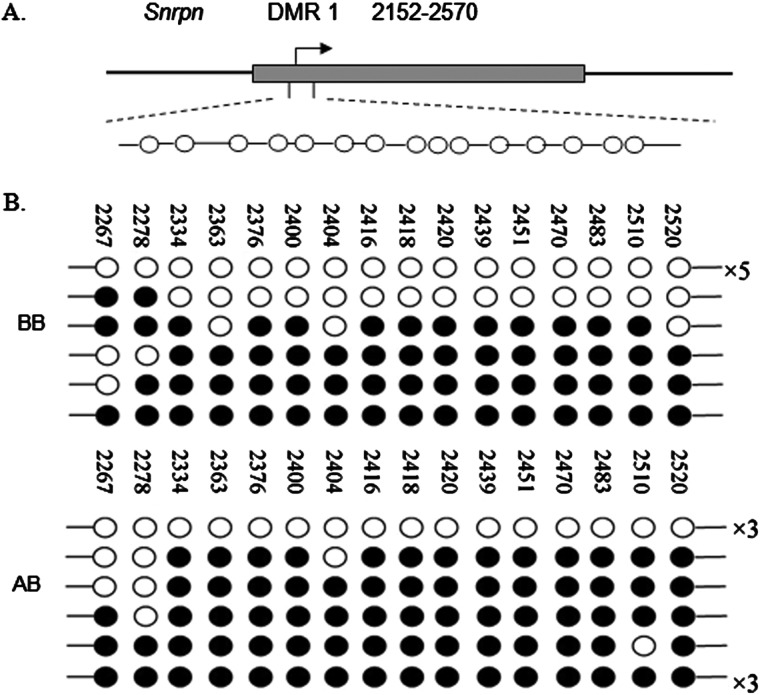
Analysis of DMR1 methylation of *Snrpn* in cultured MEFs (**A**) DNA methylation pattern of the mouse *Snrpn*. DNA methylation pattern of the mouse *Snrpn*. Arrows, *Snrpn* transcription start sites; boxes, DMR1 of the gene examined (GenBank accession no. AF081460). (**B**) DNA methylation analysis of BB and AB cells (MEFs cultured in DMEM/F12 medium and in BIX-01294-containing medium respectively) by bisulfite PCR and sequencing. Each line represents an individual clone and each circle represents a CpG nucleoside; the filled and open circles indicate hypermethylated and hypomethylated CpGs respectively. The numbers with ‘×’ given at the right-hand side of the clone lines represent the number of individual clones that show the same pattern of DNA methylation.

## DISCUSSION

EHMT2, which is mainly responsible for the monomethylation and dimethylation of H3K9 in euchromatin, is essential for early embryonic development and is involved in the transcriptional silencing of developmentally-regulated genes [20]. The EHMT2-specific inhibitor, BIX-01294, significantly reduces H3K9me2 levels in the chromatin regions. BIX-01294 was found to inhibit EHMT1 as well or better than EHMT2 [5]. Several reports [7–9] indicated that BIX-01294 reduced global H3K9me2 levels in cultured cells or embryos, but it was not appropriate for SCNT because of its high cellular toxicity. In the present study, we first identified a concentration that exerted significant effects on H3K9me2 levels in MEFs with almost no cytotoxicity. BIX-01294 was found to produce mild cytotoxic effects in MEFs treated at 2.0 μM based on morphological assessment and evaluation of cell growth (RGR) by MTT assays. Furthermore, immunocytochemical analysis using confocal microscopy showed that 1.3 μM or lower BIX-01294 produced negligible cytotoxicity and 1.3 μM was selected as the maximum concentration for further analysing its effects on epigenetic control.

Epigenetic effects, particularly those associated with imprinted genes, play a critical role in the development and function of the placenta, which in turn, plays a central role in the regulation of fetal growth and development [21]. Imprinted genes are a class of mammalian genes with possible mechanistic relationships to methylated promoters in the corresponding regions with a high frequency of CpG sites (CpG islands). DNA methylation, specifically CpG methylation of promoters, plays a crucial role in regulating gene expression. Promoter CpG methylation is important for the expression of growth factors and their receptors, cytokines and various other molecules during normal myeloid development [13].

Epigenetic regulation at imprinting control regions involves not only DNA methylation of DMRs, but also specialized changes in chromatin in the form of histone modification [15,22]. Most investigations focus on demonstrating that modifications to nuclear histone proteins alter the configuration of chromatin, which plays critical roles in transcriptional activation and repression. Histone methylation was once thought to be a stable modification, but recently it has been recognized as a dynamically regulated process catalysed by both histone methyltransferases and demethylases [23]. Previous work has shown that methylation of histone H3 at lysine 4 (H3K4me) is a mark of active genes, whereas methylation of histone H3 at lysine 9 (H3K9me) has been correlated with gene repression [24]. In the present study, using ChIP, an *in situ* technique that offers a more physiological representation of nuclear events, we studied the association of histone methylation with specific DNA sequences, to further understand the role of histone H3K9me2 in transcription.

Western blot analysis showed that global levels of H3K9me2 in MEFs treated with and without BIX-01294 (AB and BB cells respectively) were reduced by more than 50% in AB cells, compared with those in BB cells, which is consistent with previous reports [8]; But immunofluorescence staining showed weak signal change, the difference of antibodies used in immunofluorescence staining and in western blot may partly affect this much difference. However, *Snrpn* transcript levels were reduced, although mildly. Expression of specific genes is not only affected by the epigenetic levels of the whole genome but also by the surrounding epigenetic modifications. ChIP assays also showed that H3K9me2 levels of *Snrpn* were significantly reduced compared with those in BB cells. BIX-01294-induced decreased level of H3K9me2 is via inhibition of EHMT2 activity [25] and several reports have described the EHMT2-mediated gene silencing [6,8,26]. However in the present study, it seems that the EHMT2-mediated H3K9me2 down-regulation may be not sufficient for explaining the decreased expression of *Snrpn.* One group has shown the EHMT2 activation of the *β^maj^-globin* gene but independent of EHMT2 methyltransferase (HMTase) activity [27]. Therefore, we hypothesized that other epigenetic modifications, such as DNA methylation in the DMRs of *Snrpn*, might be relevant.

It has been proposed that DMR1 is associated with imprinted *Snrpn* expression [19]. *Snrpn* has a differentially methylated CpG island, DMR1, in the 5′-region, which has been shown to control imprinting of this gene. The correlation between mutations within the *Snrpn* DMR1 and the pathogenesis of the neurodevelopmental disorder PWS/AS has been extensively studied in mice and humans [28]. Furthermore, CpG methylation of promoters clearly plays a crucial role in regulating gene expression through the recruitment of repressive protein complexes and specific patterns of histone modification [29]. In the present study, we chose an area within the 6-kb 5′-DMR1 that includes a portion of the promoter and the *Snrpn* transcription start for methylation analysis [19]. A total of 16 CpG sites in the *Snrpn* DMR1 were analysed in bisulfite mutagenesis assays. Our results showed increased DNA methylation in AB cells compared with those in BB cells (66% compared with 38% respectively). The increased methylation at *Snrpn* DMR1 may partly explain the reduced *Snrpn* transcription, which is supported by previous reports [15]. Studies by Yang et al. [26] also demonstrated the significantly increased levels of global methylation by BIX-01294 in fetal pulmonary artery smooth muscle cells [26].

Our results suggest a mechanistic link between DNA and histone methylation, however the mechanism has not yet been fully elucidated. H3K9m2 is correlated with DNA methylation and is associated with transposons and repetitive sequences. Results showed that the maintenance of CpG methylation of the imprinting regulatory regions required the function of HMTase EHMT2 [11,30], methylation of H3K9 led to methylation of DNA. However other groups found that global DNA methylation levels increased significantly following BIX-01294 treatment [25,26]. Epigenetic modifier BIX-01294 may offer the prospect of ‘reverse chromatin remodelling and re-distribute the methylation pattern such as demethylation of some promoter regions, increasing methylation in repetitive DNA sequences [26]. And the present study also suggests that BIX-01294 induced an increase in DNA methylation at *Snrpn* DMR1. BIX-01294 inhibits EHMT1 as well or better than EHMT2 enzymatic activity, furthermore it is able to inhibit non-histone substrate, including DNA methyltransferase (DNMT1) [5]. BIX-01294 inhibits the methylation of DNMT1 protein by EHMT1 and EHMT2, which adds one more dimension to the complex relationship between DNA methylation and H3K9 methylation. Demethylation of DNMT1 protein by lysine-specific demethylase 1 (LSD1) enhances its stability and increases levels of DNA methylation in embryonic stem cells [31]. Biochemical and functional studies have also indicated that EHMT2 itself is capable of bringing about methylation through its ankyrin (ANK) domain independently of its HMT activity [32,33]. Furthermore, EHMT2 can probably recruit other chromatin-modifying enzymes and has been shown to affect DNA methylation [6]. The complex of DNMT1 and EHMT2 co-localizes with H3K9me2 at replication foci and leads to enhanced DNA [34]. DNMT1 and EHMT2 provide a mechanism of co-ordinated DNA and H3K9 methylation during cell division.

In addition, we speculate that other histone modifications, such as histone H3 deacetylation and H3K27 methylation, are involved in transcriptional repression. It is conceivable that in a more compromised background, such as impaired EHMT1/EHMT2 function through BIX-01294 would have a more profound effect on reversal of epigenetic control in synergy with histone deacetylases (HDACs) and DNMT inhibitors [6]. Previous studies have demonstrated that specific HDACs can be recruited locally by the maintenance methyltransferase DNMT1 [35]. More future research will be designed for detecting the molecular mechanisms underlying this epigenetic control.

Advances in assisted reproductive technology and cloning have led to a heightened interest in genomic imprinting and epigenetic reprogramming [36]. Studies suggest that EHMT2-mediated gene silencing might be a major barrier to embryonic reprogramming [3]. It has been shown that BIX-01294 can improve reprogramming efficiency in generating iPSCs from a general cell-type like MEFs [7]. A combination of four transcription factors, Oct4/sox2/Klf4/c-Myc, induce the generation of pluripotent stem cells from somatic cells [37]. BIX-01294 seems to compensate for the lack of octamer binding transcription factor 4 (Oct4) overexpression and can be speculated to function by facilitating a shift in the epigenetic balance in pluripotent gene activity from a silenced state to an active transcription state for cell reprogramming. Most cloned animals have epigenetic defects and our previous study showed better development in cloned embryos with fewer epigenetic defects [38]. SCNT cells consistently display aberrant H3K9me2 hypermethylation [1] and undergo incomplete Oct4 reactivation [39]; thus, it can be speculated that the developmental potential of SCNT embryos can be improved by specifically inhibiting EHMT2 activity with BIX-01294.

## CONCLUSIONS

Taken together, our findings indicate that BIX-01294 has important biological effects and that epigenetic regulation at imprinting control regions is highly complex. Our results suggest that BIX-01294 may reduce global levels of H3K9me2 and affect epigenetic modifications of *Snrpn* in MEFs.

## Abbreviations

AS: Angelman syndrome
BIX-01294: a diazepin-quinazolin-amine derivative
CpG: cytosine-phosphate-guanosine
DMEM: Dulbecco's modified Eagle medium
DMR: differentially methylated region
DNMT: DNA methyltransferase
EHMT2: euchromatic histone-lysine *N*-methyltransferase 2
H3K9me2: histone H3 lysine 9 dimethylation
HDAC: histone deacetylase
HMTase: EHMT2 methyltransferase
iPSC: induction of pluripotent stem cell
MEF: mouse embryonic fibroblast
NC: nitrocellulose filter
Oct4: octamer binding transcription factor 4
PWS: Prader–Willi syndrome
PWS-IC: Prader–Willi syndrome imprinting centres
RGR: relative growth rate
RT: room temperature
RT-PCR: reverse transcription PCR
SCNT: somatic cell nuclear transfer
*Snrpn*: the imprinted genes small nuclear ribonucleoprotein N

## References

[1] Wu X., Li Y., Xue L., Wang L., Yue Y., Li K., Bou S., Li G.P., Yu H. (2011). Multiple histone site epigenetic modifications in nuclear transfer and *in vitro* fertilized bovine embryos. Zygote.

[2] Chen J., Liu H., Liu J., Qi J., Wei B., Yang J., Liang H., Chen Y., Chen J., Wu Y. (2013). H3K9 methylation is a barrier during somatic cell reprogramming into iPSCs. Nat. Genet..

[3] Sridharan R., Gonzales-Cope M., Chronis C., Bonora G., McKee R., Huang C., Patel S., Lopez D., Mishra N., Pellegrini M. (2013). Proteomic and genomic approaches reveal critical functions of H3K9 methylation and heterochromatin protein-1gamma in reprogramming to pluripotency. Nat. Cell Biol..

[4] Kind J., Pagie L., Ortabozkoyun H., Boyle S., de Vries Sandra S., Janssen H., Amendola M., Nolen Leisha D., Bickmore Wendy A., van Steensel B. (2013). Single-cell dynamics of genome-nuclear lamina interactions. Cell.

[5] Chang Y., Zhang X., Horton J.R., Upadhyay A.K., Spannhoff A., Liu J., Snyder J.P., Bedford M.T., Cheng X. (2009). Structural basis for G9a-like protein lysine methyltransferase inhibition by BIX-01294. Nat. Struct. Mol. Biol..

[6] Kubicek S., O'Sullivan R.J., August E.M., Hickey E.R., Zhang Q., Teodoro M.L., Rea S., Mechtler K., Kowalski J.A., Homon C.A. (2007). Reversal of H3K9me2 by a small-molecule inhibitor for the G9a histone methyltransferase. Mol. Cell.

[7] Shi Y., Desponts C., Do J.T., Hahm H.S., Scholer H.R., Ding S. (2008). Induction of pluripotent stem cells from mouse embryonic fibroblasts by Oct4 and Klf4 with small-molecule compounds. Cell Stem Cell.

[8] Fu L., Zhang J., Yan F.X., Guan H., An X.R., Hou J. (2012). Abnormal histone H3K9 dimethylation but normal dimethyltransferase EHMT2 expression in cloned sheep embryos. Theriogenology.

[9] Fu L., Yan F.X., An X.R., Hou J. (2014). Effects of the histone methyltransferase inhibitor UNC0638 on histone H3K9 dimethylation of cultured ovine somatic cells and development of resulting early cloned embryos. Reprod. Domest. Anim..

[10] Talkowski M.E., Rosenfeld J.A., Blumenthal I., Pillalamarri V., Chiang C., Heilbut A., Ernst C., Hanscom C., Rossin E., Lindgren A.M. (2012). Sequencing chromosomal abnormalities reveals neurodevelopmental loci that confer risk across diagnostic boundaries. Cell.

[11] Schumacher A., Doerfler W. (2004). Influence of *in vitro* manipulation on the stability of methylation patterns in the Snurf/Snrpn-imprinting region in mouse embryonic stem cells. Nucleic Acids Res..

[12] Shemer R., Birger Y., Riggs A.D., Razin A. (1997). Structure of the imprinted mouse Snrpn gene and establishment of its parental-specific methylation pattern. Proc. Natl. Acad. Sci. U.S.A..

[13] Benetatos L., Hatzimichael E., Dasoula A., Dranitsaris G., Tsiara S., Syrrou M., Georgiou I., Bourantas K.L. (2010). CpG methylation analysis of the MEG3 and SNRPN imprinted genes in acute myeloid leukemia and myelodysplastic syndromes. Leuk. Res..

[14] Tran D.A., Bai A.Y., Singh P., Wu X., Szabo P.E. (2014). Characterization of the imprinting signature of mouse embryo fibroblasts by RNA deep sequencing. Nucleic Acids Res.

[15] Yao J., Huang Y., Huang R., Shi R., Chen P., Zhu B., Li M., Jiang X., Zheng M., Jiang Y., Yang X. (2012). Epigenetic modifications and mRNA levels of the imprinted gene Grb10 in serially passaged fibroblast cells. Biochimie.

[16] Lu G., Cui S.J., Geng X., Ye L., Chen B., Feng Z.G., Zhang J., Li Z.Z. (2013). Design and preparation of polyurethane-collagen/heparin-conjugated polycaprolactone double-layer bionic small-diameter vascular graft and its preliminary animal tests. Chin. Med. J..

[17] Schoppee Bortz P.D., Wamhoff B.R. (2011). Chromatin immunoprecipitation (ChIP): revisiting the efficacy of sample preparation, sonication, quantification of sheared DNA, and analysis via PCR. PLoS One.

[18] Pharmacopeia U.S., The United States Pharmacopeial Convention (2004). Biological reactivity test, *in vitro*. USP Pharmacists' Pharmacopeia.

[19] Miyazaki K., Mapendano C.K., Fuchigami T., Kondo S., Ohta T., Kinoshita A., Tsukamoto K., Yoshiura K., Niikawa N., Kishino T. (2009). Developmentally dynamic changes of DNA methylation in the mouse Snurf/Snrpn gene. Gene.

[20] Park K.E., Johnson C.M., Wang X., Cabot R.A. (2011). Differential developmental requirements for individual histone H3K9 methyltransferases in cleavage-stage porcine embryos. Reprod. Fertil. Dev..

[21] Yamaguchi S., Shen L., Liu Y., Sendler D., Zhang Y. (2013). Role of Tet1 in erasure of genomic imprinting. Nature.

[22] Feng S., Jacobsen S.E., Reik W. (2010). Epigenetic reprogramming in plant and animal development. Science.

[23] Xu W., Li Z., Yu B., He X., Shi J., Zhou R., Liu D., Wu Z. (2013). Effects of DNMT1 and HDAC inhibitors on gene-specific methylation reprogramming during porcine somatic cell nuclear transfer. PLoS One.

[24] Schwammle V., Jensen O.N. (2013). A computational model for histone mark propagation reproduces the distribution of heterochromatin in different human cell types. PLoS One.

[25] Lu Z., Tian Y., Salwen H.R., Chlenski A., Godley L.A., Raj J.U., Yang Q. (2013). Histone-lysine methyltransferase EHMT2 is involved in proliferation, apoptosis, cell invasion, and DNA methylation of human neuroblastoma cells. Anticancer Drugs.

[26] Yang Q., Lu Z., Singh D., Raj J.U. (2012). BIX-01294 treatment blocks cell proliferation, migration and contractility in ovine foetal pulmonary arterial smooth muscle cells. Cell Prolif.

[27] Chaturvedi C.P., Hosey A.M., Palii C., Perez-Iratxeta C., Nakatani Y., Ranish J.A., Dilworth F.J., Brand M. (2009). Dual role for the methyltransferase G9a in the maintenance of beta-globin gene transcription in adult erythroid cells. Proc. Natl. Acad. Sci. U.S.A..

[28] Wu M.Y., Jiang M., Zhai X., Beaudet A.L., Wu R.C. (2012). An unexpected function of the Prader-Willi syndrome imprinting center in maternal imprinting in mice. PLoS One.

[29] Piedrahita J.A. (2011). The role of imprinted genes in fetal growth abnormalities. Birth Defects Res A. Clin. Mol. Teratol..

[30] Xin Z., Tachibana M., Guggiari M., Heard E., Shinkai Y., Wagstaff J. (2003). Role of histone methyltransferase G9a in CpG methylation of the Prader-Willi syndrome imprinting center. J. Biol. Chem..

[31] Lanouette S., Mongeon V., Figeys D., Couture J.F. (2014). The functional diversity of protein lysine methylation. Mol. Syst. Biol..

[32] Epsztejn-Litman S., Feldman N., Abu-Remaileh M., Shufaro Y., Gerson A., Ueda J., Deplus R., Fuks F., Shinkai Y., Cedar H., Bergman Y. (2008). *De novo* DNA methylation promoted by G9a prevents reprogramming of embryonically silenced genes. Nat. Struct. Mol. Biol..

[33] Dong K.B., Maksakova I.A., Mohn F., Leung D., Appanah R., Lee S., Yang H.W., Lam L.L., Mager D.L., Schubeler D. (2008). DNA methylation in ES cells requires the lysine methyltransferase G9a but not its catalytic activity. EMBO J.

[34] Esteve P.O., Chin H.G., Smallwood A., Feehery G.R., Gangisetty O., Karpf A.R., Carey M.F., Pradhan S. (2006). Direct interaction between DNMT1 and G9a coordinates DNA and histone methylation during replication. Genes Dev.

[35] Gu Y., Yang P., Shao Q., Liu X., Xia S., Zhang M., Xu H., Shao Q. (2013). Investigation of the expression patterns and correlation of DNA methyltransferases and class I histone deacetylases in ovarian cancer tissues. Oncol. Lett..

[36] de Waal E., Yamazaki Y., Ingale P., Bartolomei M., Yanagimachi R., McCarrey J.R. (2012). Primary epimutations introduced during intracytoplasmic sperm injection (ICSI) are corrected by germline-specific epigenetic reprogramming. Proc. Natl. Acad. Sci. U.S.A..

[37] Lou X.X., Nakagawa T., Nishimura K., Ohnishi H., Yamamoto N., Sakamoto T., Ito J. (2013). Reprogramming of mouse cochlear cells by transcription factors to generate induced pluripotent stem cells. Cell Reprogram.

[38] Yang X.Y., Li H., Ma Q.W., Yan J.B., Zhao J.G., Li H.W., Shen H.Q., Liu H.F., Huang Y., Huang S.Z. (2006). Improved efficiency of bovine cloning by autologous somatic cell nuclear transfer. Reproduction.

[39] Pfeiffer M.J., Esteves T.C., Balbach S.T., Arauzo-Bravo M.J., Stehling M., Jauch A., Houghton F.D., Schwarzer C., Boiani M. (2013). Reprogramming of two somatic nuclei in the same ooplasm leads to pluripotent embryonic stem cells. Stem Cells.

